# Cyclophilin A secreted from fibroblast-like synoviocytes is involved in the induction of CD147 expression in macrophages of mice with collagen-induced arthritis

**DOI:** 10.1186/1476-9255-9-44

**Published:** 2012-11-20

**Authors:** Tsuyoshi Nishioku, Shinya Dohgu, Mitsuhisa Koga, Takashi Machida, Takuya Watanabe, Teppei Miura, Kousuke Tsumagari, Mariko Terasawa, Atsushi Yamauchi, Yasufumi Kataoka

**Affiliations:** 1 Department of Pharmaceutical Care and Health Sciences, Faculty of Pharmaceutical Sciences, Fukuoka University, Fukuoka, 8-19-1 Nanakuma, Jonan-ku, Fukuoka, 814-0180, Japan

**Keywords:** CD147, Collagen-induced arthritis, Cyclophilin A, Macrophage, Rheumatoid arthritis

## Abstract

**Background:**

Cyclophilin A (CypA), a member of the immunophilin family, is a ubiquitously distributed intracellular protein. Recent studies have shown that CypA is secreted by cells in response to inflammatory stimuli. Elevated levels of extracellular CypA and its receptor, CD147 have been detected in the synovium of patients with RA. However, the precise process of interaction between CypA and CD147 in the development of RA remains unclear. This study aimed to investigate CypA secretion from fibroblast-like synoviocytes (FLS) isolated from mice with collagen-induced arthritis (CIA) and CypA-induced CD147 expression in mouse macrophages.

**Findings:**

CIA was induced by immunization with type II collagen in mice. The expression and localization of CypA and CD147 was investigated by immunoblotting and immunostaining. Both CypA and CD147 were highly expressed in the joints of CIA mice. CD147 was expressed in the infiltrated macrophages in the synovium of CIA mice. *In vitro*, spontaneous CypA secretion from FLS was detected and this secretion was increased by stimulation with lipopolysaccharide. CypA markedly increased CD147 levels in macrophages.

**Conclusions:**

These findings suggest that an interaction in the synovial joints between extracellular CypA and CD147 expressed by macrophages may be involved in the mechanisms underlying the development of arthritis.

## Introduction

Rheumatoid arthritis (RA) is an autoimmune inflammatory disease characterized by synovial hyperplasia and articular cartilage degradation. The hyperplasia of the synovial lining is largely composed of increased number of fibroblast-like synoviocytes (FLS) and macrophages. FLS play a critical role in RA pathogenesis by inducing the inflammatory microenvironment in the synovial joints through production of pro-inflammatory factors or recruitment of other immune cells. Macrophages are localized in the synovial lining with FLS, where they modulate the function of each other through surface molecules as well as soluble mediators [1].

Cyclophilin A (CypA), the most abundant cytoplasmic cyclophilin, has been identified as the intracellular binding protein for the immunosuppressive drug cyclosporine A [2,3]. Although CypA was initially shown to function primarily as an intracellular protein, recent studies have shown that it can be secreted by cells in response to inflammatory stimuli [4-8]. Elevated levels of extracellular CypA have been detected in the synovial fluid of patients with RA [9]. CD147 is a cell surface glycoprotein that belongs to the immunoglobulin superfamily and was identified as the signaling receptor for extracellular CypA [10]. CD147 is also known as EMMPRIN (extracellular matrix metalloproteinase inducer), and the expression of CD147 stimulates the production of matrix metalloproteinases (MMPs) [11]. Increased expression of CD147 has been shown in patients with RA [12,13]. Recent experimental finding suggests that CypA-CD147 interactions may be associated with the recruitment of leucocytes into the joint tissues [14]. However, the precise process of interaction between CypA and its receptor CD147 in the development of RA remains unclear.

In the present study, we demonstrated that both CypA and CD147 are highly expressed in the joints of mice with collagen-induced arthritis (CIA). CypA was released from FLS, and increased CD147 expression in macrophages. These findings suggest that an interaction in the synovial joints between extracellular CypA and CD147 expressed by macrophages forms one of the critical mechanisms underlying the pathogenesis of RA.

## Methods

### Animals

Six-week-old male DBA/1J and C57BL/6N mice were purchased from Kyudo (Tosu, Japan). All the procedures involving experimental animals adhered to the law (No. 105) and notification (No. 6) of the Japanese Government, and were approved by the Laboratory Animal Care and Use Committee of Fukuoka University.

### Induction of CIA in mice

Bovine type II collagen (CII) (Chondrex) was emulsified with an equal volume of complete Freund’s adjuvant (Chondrex) to give a final concentration of 1 mg/mL. DBA/1J mice were immunized intradermally at the base of the tail with 100 μg emulsified CII. Age-matched DBA/1J mice without CIA were employed as controls. The severity of arthritis was graded on a 0–4 scale, as previously described [15]. Mice showing scale 4 in the severity of arthritis were supplied for the following experiments.

### Isolation of FLS from CIA mice

Mouse FLS were isolated from the tarsus of the hind paws of CIA mice. After careful removal of the skin, joints were minced and digested for 2 h at 37°C in Dulbecco’s modified Eagle’s medium (DMEM) containing collagenase type 4 and DNase. After filtration through a 100-μm cell strainer, cell suspension was collected, centrifuged, resuspended, and cultured in DMEM supplemented with 10% fetal bovine serum (FBS). Cultured mouse FLS at 80–90% confluency in a dish (6 cm in diameter) were used for *in vitro* assays from passage 3 to 7.

### Isolation of bone marrow-derived macrophages from mice

C57BL/6N mice were euthanized, and the femur and tibia of the hind legs were dissected. Bone marrow cavities were flushed with Minimum Essential Medium (MEM)-α. The bone marrow cells were cultured in MEM-α supplemented with 10% FBS, 20 ng/mL macrophage-colony stimulating factor (Sigma) for 5 days. Before use, bone marrow-derived macrophages were washed vigorously to remove nonadherent cells.

### Immunohistochemistry of synovial tissues of control and CIA mice

**Figure 1:**
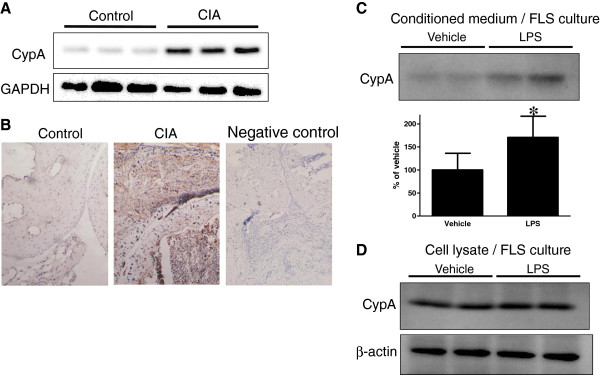
**CypA expression in synovial tissues and CypA secretion from FLS of CIA mice.** (**a**) Representative western blots of CypA levels in the lysates obtained from synovial tissues of control and CIA mice. Protein levels of CypA were normalized to GAPDH and the data are expressed as the percentage of control values. Three separate experiments (n = 3 mice in each) were performed (control: 100.0 ± 4.4%, CIA: 668.0 ± 22.24%). (**b**) Representative photographs showing immunohistochemical staining of CypA in synovial tissues of control and CIA mice (left and middle panel, respectively; n = 4 mice in each group). Right panel (negative control) showing immunohistochemical staining of CypA in synovial tissues of CIA mice according to the method described above, except that the primary antibody was omitted. (**c**,**d**) Representative western blots of CypA in the conditioned medium (c: top panel) and in the cell lysates (d) of FLS culture treated with vehicle and LPS (100 ng/mL for 24 h). β-actin was used as an internal control. Densitometric analysis was performed on protein bands and the data are expressed as the percentage of vehicle values. Each bar indicates mean ± S.E.M. (*n* = 5) *P < 0.05, significantly different from vehicle (c: bottom panel). Three separate experiments (n = 2 dishes in each) were performed (vehicle: 100.0 ± 1.0%, LPS: 104.9 ± 1.9%) (d).

**Figure 2:**
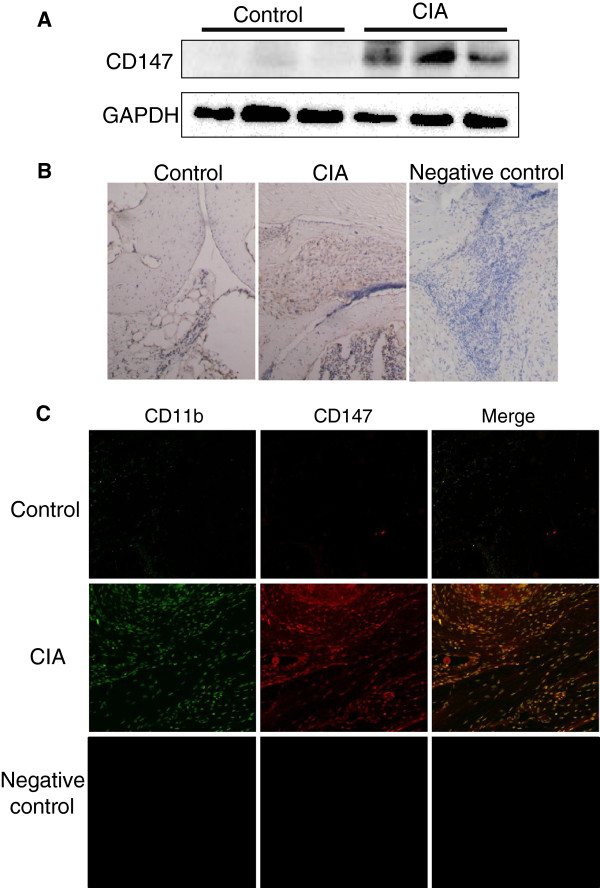
**CD147 expression in the synovial tissues of CIA mice.** (**a**) Representative western blots of CD147 levels in tissue lysates of the synovial joints of control and CIA mice. GAPDH was used as an internal control. Densitometric analysis was performed on protein bands and the data are expressed as the percentage of control values. Three separate experiments (n = 3 mice in each) were performed (control: 100.0 ± 17.4%, CIA: 1144.2 ± 125.1%). (**b**) Representative photographs showing immunohistochemical staining of CD147 in synovial tissues of control and CIA mice (left and middle panel, respectively; n = 4 mice in each group). Right panel (negative control) showing immunohistochemical staining of CD147 in synovial tissues of CIA mice according to the method described above, except that the primary antibody was omitted. (**c**) Representative photographs showing immunohistochemical staining of CD147 in infiltrated CD11b-immunopositive cells (mainly for macrophages) of synovial tissues of control and CIA mice (top and middle panel, respectively; n = 4 mice in each group). Frozen joints sections were stained with antibodies for CD11b (green) and CD147 (red) for double immunofluorescence staining. Bottom panel (negative control) showing double immunofluorescence staining of CD11b and CD147 in synovial tissues of CIA mice according to the method described above, except that the primary antibodies were omitted.

The ankle joints were fixed in 4% paraformaldehyde and decalcified until the bones were pliable. Sagittal sections were prepared on a cryostat. The sections were incubated with anti-CypA (Proteintech Group), anti-CD147 (Abcam) and anti-CD11b (Thermo Fisher Scientific) antibodies. After incubation with primary antibodies, the sections were further incubated with peroxidase-conjugated anti-rabbit IgG secondary antibody. Color was developed using the DAKO Liquid DAB + Substrate Chromogen System (Dako North America), followed by counterstaining with hematoxylin. The sections of CIA mice were stained according to the method described above, except that the primary antibody was omitted. Weak non-specific staining was observed (negative control in Figures 1b and 2b).

For double immunofluorescence staining, the sections were incubated with a combination of rat anti-CD147 (AbD Serotec) and rabbit anti-CD11b antibodies. Cy3-conjugated anti-rat IgG and fluorescein isothiocyanate (FITC)-conjugated anti-rabbit IgG antibodies were used as secondary antibodies. The sections of CIA mice were stained according to the method described above, except that the primary antibodies were omitted. Non-specific staining was almost null (negative control in Figure 2c). Images were captured using Confocal microscope (OLYMPUS).

### Immunoblot analysis of CypA and CD147

Mice were anesthetized with sodium pentobarbital and decapitated. The ankle joints were frozen with liquid nitrogen, crushed, and lysed in RIPA buffer. Mouse FLS and macrophages were washed with PBS and lysed in RIPA buffer. Conditioned media from FLS were concentrated using an Amicon Ultra-50k and 10k centrifugal filters (Millipore). Denatured lysates and concentrated conditioned media were separated by SDS-PAGE and transferred to polyvinylidene difluoride membranes. The membranes were immunoblotted with anti-CypA, anti-CD147, anti-glyceraldehyde 3-phosphate dehydrogenase (GAPDH) (EPITOMICS) and anti-β-actin (IMGENEX) antibodies. Immunoblots were then exposed to peroxidase-conjugated secondary antibodies and visualized using a SuperSignal West Femto Maximum Sensitivity Substrate (Thermo Fisher Scientific).

### Statistical analysis

Values are expressed as the mean ± S.E.M. Statistical analysis was performed using a Student’s *t* test or one way analysis of variance followed by Dunnett’s *post hoc* test. The differences between the means were considered to be significant at *P* < 0.05.

## Results and discussion

Immunoblotting and immunohistochemical analysis showed that CypA was highly expressed in the joints of CIA mice compared with control mice (Figure 1a and b, respectively). This finding is consistent with a report of Damsker et al. [14]; these support the clinical evidence that elevated levels of extracellular CypA have been found in the synovial fluids of RA patients [9]. To investigate whether FLS isolated from CIA mice secreted CypA, the conditioned media were obtained from FLS cultures and analyzed by western blotting (Figure 1c). FLS isolated from CIA mice were found to secrete CypA. CypA enhances the secretion of MMP-2 and MMP-9, cell invasion, and production of inflammatory cytokines in monocytes [16]. These findings suggest that increased CypA in the synovial fluid may have a role in RA development.

When FLS were stimulated with lipopolysaccharide (LPS), CypA secretion was significantly increased to 170.5 ± 46.2% of control FLS (vehicle). LPS had no effect on the intracellular levels of CypA in FLS (Figure 1d). Toll-like receptor 4 (TLR4), a receptor for LPS, is highly expressed in the synovial tissue of RA patients [17]. Mice defective in *Tlr4* are protected from experimental arthritis [18], and TLR4 inhibitors ameliorate destructive arthritis in mice [19]. Furthermore, endogenous TLR4 ligands, including heat shock proteins, tenascin-C, and S100 proteins, are expressed in the rheumatoid joints [20-22]. These findings suggest that endogenous TLR4 ligands may stimulate FLS to secrete CypA in CIA mice.

**Figure 3:**
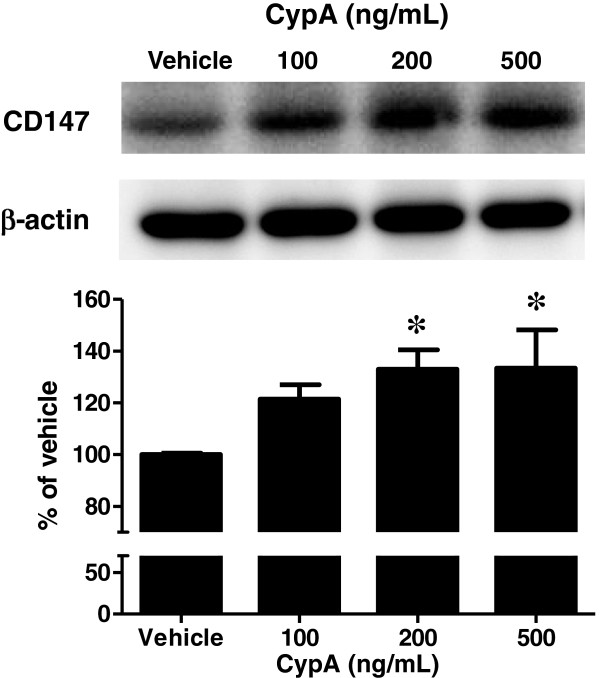
**Effect of CypA on CD147 expression by cultured mouse macrophages.** Macrophage cultures were treated with CypA (100–500 ng/mL) for 24 h. Representative western blots (top panel) and densitometric analysis (bottom panel) of CD147 in the cultured macrophages. β-actin was used as an internal control. Band intensities were quantified by scanning densitometry and the data are expressed as relative ratio of CypA treatment to vehicle treatment (vehicle). Values are the means ± S.E.M. (*n* = 7) *P < 0.05, significantly different from vehicle.

As shown in Figure 2, band intensities (a) and immunoreactivities (b) for CD147 were markedly increased in the joints of CIA mice. Elevated CD147 expression is shown in the synovial membrane of RA patients (16), and CD147 stimulates MMP production in the synovial tissue of affected joints in RA patients (17). The synovial tissues of CIA mice showed abundant staining of CD11b, mainly for macrophages (data not shown). Based on their distribution pattern and histological shape, these CD147-immunoreactive cells were identified as potentially being macrophages. We investigated the localization of CD147 in macrophages in the synovial sections using antibodies for CD147 and CD11b. The immunoreactivities for CD147 were found to co-localize with those for CD11b in CIA synovial tissues (Figure 2c). CD11b is expressed on leukocytes including macrophages, granulocytes and NK cells. In our double immunofluorescence staining of the joints of mice with CIA, co-localization of CD147 and infiltrated macrophages could not be detected by using other mouse macrophage marker (F4/80) due to the undesirable high background. The primary culture of macrophages was exposed to CypA for 24 h. Representative western blots for CD147 (top panel) and densitometry analysis for these intensities (bottom panel) showed that CypA (100, 200 and 500 ng/mL) dose-dependently increased CD147 levels by 121.5 ± 5.6, 133.0 ± 7.5 and 133.4 ± 14.8%, respectively (Figure 3). CypA is frequently used at concentrations ranging from 100 to 1000 ng/mL. In the present study, CypA (200 and 500 ng/mL) increased CD147 expression in macrophages. The concentration of CypA employed in the current study is within the range frequently used in *in vitro* studies, although we did not determine the concentration of CypA in the joints of CIA mice. CypA-induced CD147 expression in macrophages is an entirely novel finding in the present study. There was a considerable increase in CD147 expression in CIA synovial tissue (Figure 2c). However, the effect of CypA on macrophages was modest, with an approximate maximum increase of 30% (Figure 3). Therefore, it is likely that the increase in CD147 expression in infiltrated macrophages of synovial tissues of CIA mice (Figure 2c) may have been mediated by several endogenous mediators, including CypA. It has been known that CypA is a paracrine and autocrine modulator of endothelial cell function in vascular disease [5]. CD147 expression in monocytes/macrophages in RA synovium is much higher than that in those cells derived from peripheral blood of RA patients [23]. We used two strains of mice in the current study. DBA/1J mice are well known to be the most sensitive to the induction of CIA. C57BL/6N mice were used for the experiment with isolated bone marrow-derived macrophages because of the convenient purchase and cost in our university. As shown in Figure 1, CypA expression in synovial tissues and CypA secretion from FLS were observed in CIA mice (DBA/1J strain). These mice showed increased expression of CD147 in infiltrated macrophages of synovial tissues. Therefore, our findings strongly suggest that CypA is required for increased expression of CD147 in macrophages isolated from DBA/1J mice in a similar manner to C57BL/6N mice (Figure 3). These findings suggest that CypA secreted from FLS stimulates macrophages to express CD147 in the RA synovium. Therefore, an interaction in the synovial joints between extracellular CypA and CD147 on macrophages may be involved in the mechanisms underlying the development of arthritis. Regulation of CypA secretion and CD147 expression in the synovial joints could be potential therapeutic targets for RA.

## Abbreviations

CII: Bovine type II collagen; CIA: Collagen-induced arthritis; CypA: Cyclophilin A; DMEM: Dulbecco’s modified Eagle’s medium; FLS: Fibroblast-like synoviocytes; GAPDH: Glyceraldehyde 3-phosphate dehydrogenase; LPS: Lipopolysaccharide; MEM: Minimum Essential Medium; MMP: Matrix metalloprotienase; RA: Rheumatoid arthritis.

## Competing interests

The authors declare that they have no competing interests.

## Authors' contributions

TN: Conceived, designed and conducted experiments for the study and wrote the manuscript. TM, KT, MT: Performed experiments and helped in constructing the figures. MK, TW, TM: Participated in the study design and coordination and drafting of the manuscript. SD, AY: Contributed to supervision of laboratory procedures, data analysis and interpretation. YK: Conceived and designed the study, contributed to data analysis and interpretation and wrote the manuscript. All authors have read and approved this manuscript.
